# Correlation between single nucleotide polymorphisms of folate metabolism genes and ethnic distribution in pregnant women

**DOI:** 10.1097/MD.0000000000034472

**Published:** 2023-07-28

**Authors:** Hua Huang, Jiangyan He, Dongyang Deng, Rong Chen, Yiyuan Zhou

**Affiliations:** a College of Clinical Medicine, Guizhou University of Traditional Chinese Medicine, Guiyang, China; b Maternity Department, the First Affiliated Hospital of Guizhou University of Traditional Chinese Medicine, Guiyang, China; c Eugenics Research Center, the First Affiliated Hospital of Guizhou University of Traditional Chinese Medicine, Guiyang, China.

**Keywords:** 10-methylenetetrahydrofolate reductase, 5, folate metabolism, methionine synthase reductase, pregnant women, single nucleotide polymorphism

## Abstract

This retrospective study aims to identify the single nucleotide polymorphisms (SNPs) of 5,10-methylenetetrahydrofolate reductase (MTHFR) (C677T, A1298C), methionine synthase reductase (MTRR) (A66G) and ethnic distribution characteristics in pregnant women, and to explore the risk correlation with folate metabolism. The demographic data of 8735 pregnant women aged 15 to 47 years were retrospectively analyzed, and peripheral blood samples were collected and tested. Reverse transcription-quantitative polymerase chain reaction was applied to determine the genotype and allele frequency of MTHFR C677T, A1298C and MTRR A66G in blood samples. Sperman correlation analysis, univariate and multivariate logistic regression analysis were used to verify the correlation between SNPs of MTHFR (C677T, A1298C), MTRR (A66G), different ethnic groups and the susceptibility and risk levels of folate metabolism. The relative risk of the SNPs was further determined by calculating the odds ratio (OR) at a 95% confidence interval (CI). The average age of 8735 pregnant women was 28.87 ± 4.20 years old. The evaluation of risk levels for folate metabolism was relative high, including 2296 cases with low risk, 3971 cases with medium risk, and 752 cases with high risk. Among the MTHFR C677T locus, the CC genotype had the highest frequency, MTHFR A1298C locus had the highest frequency of the AA genotype, and MTRR A66G locus had the highest frequency of the AA genotype. The frequency distribution of SNPs in different ethnic groups revealed that the frequency of CT genotype among the MTHFR C677T locus, AA genotype among the MTHFR A1298C locus and the MTRR A66G locus was the highest in Han, Buyi, Miao and Dong ethnic groups. The results of logistic regression analysis showed that the Han, Buyi, Miao and other ethnic groups (including Yi, Bai, Zhuang, Chuanqing) had the possibility of increasing the risk levels of folate metabolism. The CC genotype of MTHFR C677T (adjusted OR = 2.46, 95% CI: 2.14–2.84, *P* < .001) and the AG genotype of MTRR A66G (adjusted OR = 1.89, 95% CI: 1.61–2.22, *P* < .001) were significantly related to the risk levels of folate metabolism, which is an independent risk factor for the susceptibility of folate metabolism.

## 1. Introduction

Folate (vitamin B9) refers to a group of water-soluble compounds that plays a crucial role in the growth of body cells and embryonic development. It participates in numerous 1-carbon transfer reactions, including purine and pyrimidine biosynthesis, amino acid interconversions and DNA methylation.^[1]^ These whole biochemical processes have a significant influence on hematopoietic, cardiovascular and nervous system functions. The disturbances of the folate cycle can result in neural tube defects (NTDs) in babies, chronic hypertension, coronary artery disease, a higher risk of heart infarction, cancer development, and psychic and neurodegenerative disorders.^[2]^ Furthermore, pregnant women with folate deficiency are more likely to have hyperhomocysteinemia than those without folate deficiency.^[3]^ However, the human body cannot synthesize folic acid, and can only rely on exogenous supply. After absorption, the body has a short storage time and needs to be supplemented continuously. Although the World Health Organization and National Health Commission of the People Republic of China have developed relevant guidelines for folic acid supplementation in pregnancy, the levels of folic acid awareness, knowledge and use among Chinese women living in different area during early pregnancy are very low and folate deficiency was quite prevalent.^[4]^

Folic acid deficiency is usually caused by insufficient intake of folic acid as well as an insufficient ability to metabolize folic acid due to individual genetic causes.^[5]^ The complex process of folate metabolism requires adequate activity of many enzymes and presence of co-enzymes.^[6]^ The key enzymes in folate metabolism are 5,10-methylenetetrahydrofolate reductase (MTHFR) and methionine synthase reductase (MTRR). The polymorphisms rs1801394 (A66G) of the MTRR gene and rs1801133 (C677T), rs1801131 (A1298C) of the MTHFR are important regulatory genes in the process of folic acid metabolism. Among them, the allele C of the MTHFR gene is frequently replaced by allele T, changing alanine into valine. This mutation will produce CC (wild type), CT (heterozygous mutant) and TT (homozygous mutant) polymorphisms and lower enzymatic activity of folate metabolism.^[7]^ Several studies have previously analyzed the associations between the nucleotide polymorphisms (SNPs) of MTHFR and MTRR gene in folate metabolism. Among them, many studies have indicated polymorphisms C677T, A1298C of the MTHFR gene and A66G of the MTRR gene were associated with lower enzymatic activity of folate metabolism and fetus defects.^[8–10]^ However, in another study, MTHFR (C677T, A1298C) and MTRR A66G polymorphisms were found to be protector factors for NTDs in the mother group.^[11]^ Obviously, the associations between the SNPs of MTHFR and MTRR gene with the susceptibility of folate metabolism were still not completely revealed and needed to be identified in the large sample clinical studies.

Up to now, there are few studys on the polymorphisms and ethnic distribution of MTHFR (C677T, A1298C) and MTRR (A66G) in our region (Guiyang, Guizhou Province), which cannot provide credible evidence for the recommendations for folic acid supplementation during pregnancy. In this study, we aimed to identify the distribution characteristics of SNPs among different ethnic groups, and to explore the associations between the SNPs of MTHFR (C677T, A1298C) and MTRR (A66G) with the risk correlation with folate metabolism in pregnant women.

## 2. Materials and methods

### 2.1. Study objects

A retrospective study was carried out in Guiyang. During September 2020 to August 2021, all clinical data of pregnant women were retrospectively analyzed. The eligibility criteria of this study were as follows: inclusion criteria: pregnant women with gestational week ≥10; singleton pregnancy; voluntary for SNPs of folate metabolism testing. Exclusion criteria: pregnant women with history of habitual abortion, metabolic diseases (diabetes, hyperthyroidism) or metabolic syndrome and thrombophilia; clinical data were missing or unavailable. Totally, 8735 pregnant women (aged 15–47 years old) who came to the hospital for folic acid metabolism tests were selected as the study subjects. All study subjects provided written informed consent for SNPs testing, and their blood samples were collected for tests. This study was approved by the ethics committee of the First Affiliated Hospital of Guizhou University of Traditional Chinese Medicine (number: K2022-011, Supplement 1, http://links.lww.com/MD/J381).

### 2.2. Samples collection and DNA extraction

Three milliliter of blood samples were collected from each subject, and was placed in an anticoagulant tube containing ethylenediaminetetraacetic acid. DNA was extracted using the whole blood genomic DNA extraction kit (Kangwei Shiji Biotechnology Co., Ltd., Beijing, Cat No.: CW2516), The concentration, purity, and quality of extracted DNA were examined by UV spectrophotometer, and only qualified samples were stored in −20°C refrigerator for subsequent experiments.

### 2.3. Gene polymorphisms analysis

Gene polymorphisms detection kit (Hechuan Biotechnology Co., Ltd., Hangzhou, Cat No.: KBS-1016-017) and reverse transcription-quantitative polymerase chain reaction (RT-qPCR) were applied to the detection of SNPs among MTHFR (C677T, A1298C) and MTRR A66G locus. RT-qPCR were performed on an ABI7900 real-time PCR instrument. Conditions of RT-qPCR were: pre-denaturation, 94°C for 10 minutes; denaturation, 94°C for 20 seconds, annealing and extension, 57°C, amplification for 39 cycles; collect and read fluorescence, 30°C for 1 minute. The genotype results of each sample were determined using the analysis software of the PCR machine.

### 2.4. Statistical analysis

Excel software (version: 2016, Microsoft) was used to record all data. SPSS 25.0 software (IBM Inc., Chicago) was used to analyze the frequency and carrier rate of genetypes, and the enumeration data results were expressed as rate (%). Goodness-of-fit test was used to determine whether the genotype frequency distribution conformed to Hardy–Weinberg equilibrium (HWE), and spearman correlation analysis or logistic regression analysis (unadjusted and adjusted) was used to analyze the correlation between variables.

## 3. Results

### 3.1. Demographic information of pregnant women

**Figure 1.**
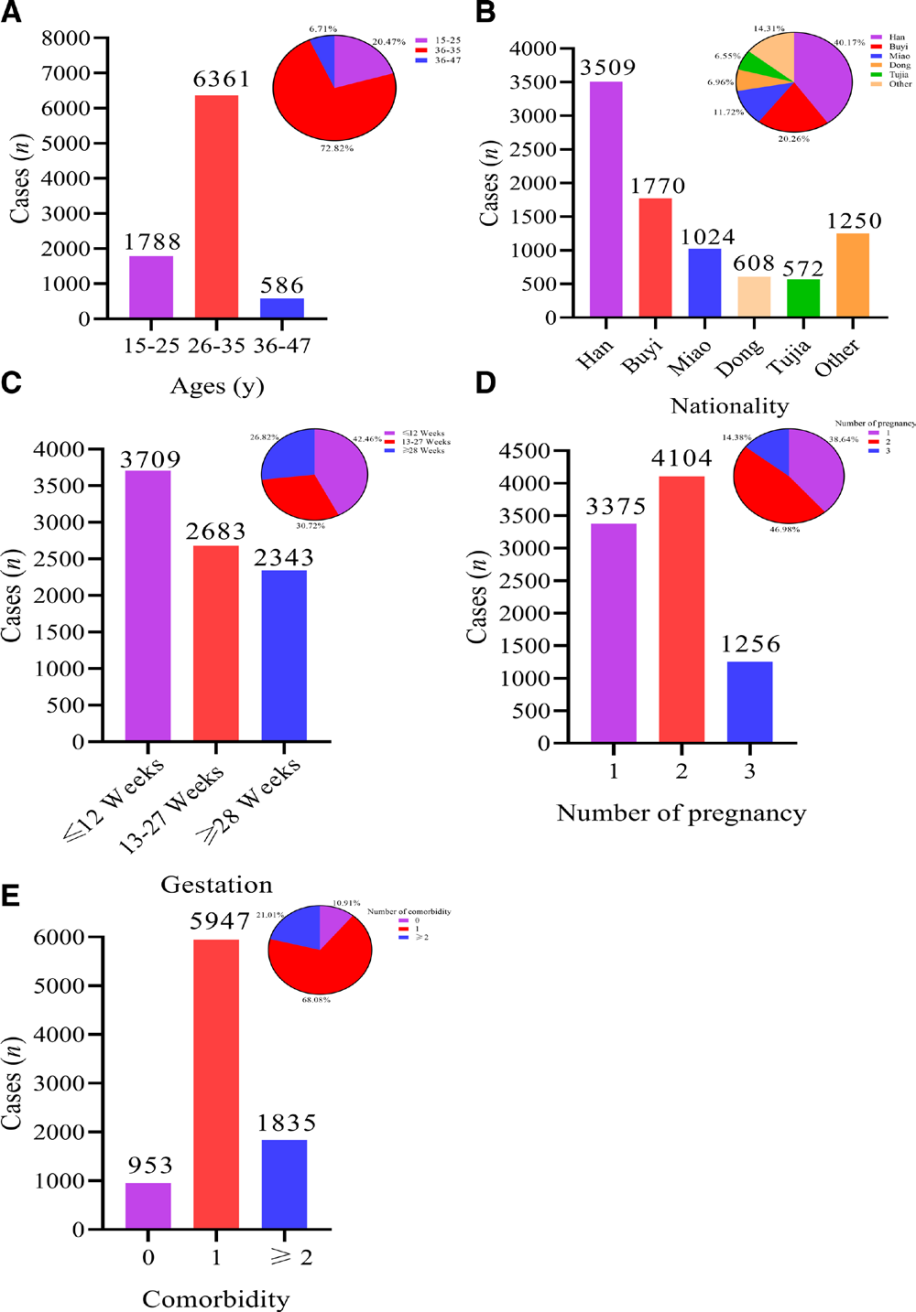
Demographic information of 8735 pregnant women. (A) Age distribution of included cases; (B) Ethnic distribution of included cases; (C) Gestational distribution of included cases; (D) Number of pregnancies of included cases; (E) Pregnancy complications of included case.

A total of 8735 pregnant women were included in this cohort study, with an average age of 28.87 ± 4.20 years, including 1788 women aged 15 to 25, 6361 women aged 26 to 35 and 586 women aged 36 to 47 (Fig. 1A). In the ethnic distribution, there are 3509 cases of Han nationality, 1770 cases of Buyi nationality, 1024 cases of Miao nationality, 608 cases in the Dong ethnic group, 572 cases in the Tujia ethnic group, and 1250 cases in other ethnic groups (including the Yi, Bai, Zhuang, and Chuanqing ethnic groups) (Fig. 1B). In the gestational distribution, there were 3709 cases in the first trimester (≤12 weeks), 2683 cases in the second trimester (13–27 weeks), and 2343 cases in the third trimester (≥28 weeks) (Fig. 1C). In the statistics of the number of pregnancies, there were 3375 cases of first pregnancy, 4104 cases of second pregnancy, and 1256 cases of third pregnancy (Fig. 1D). Among the complications during pregnancy, there were 953 cases without complications during pregnancy, a total of 5947 cases with one of the complications (gestational hypertension, gestational diabetes mellitus, hyperthyroidism during pregnancy, obesity, infectious diseases, scarred uterus, etc), and 1835 cases with complications ≥2 (Fig. 1E).

### 3.2. SNPs and risk levels of folate metabolism

**Figure 2.**
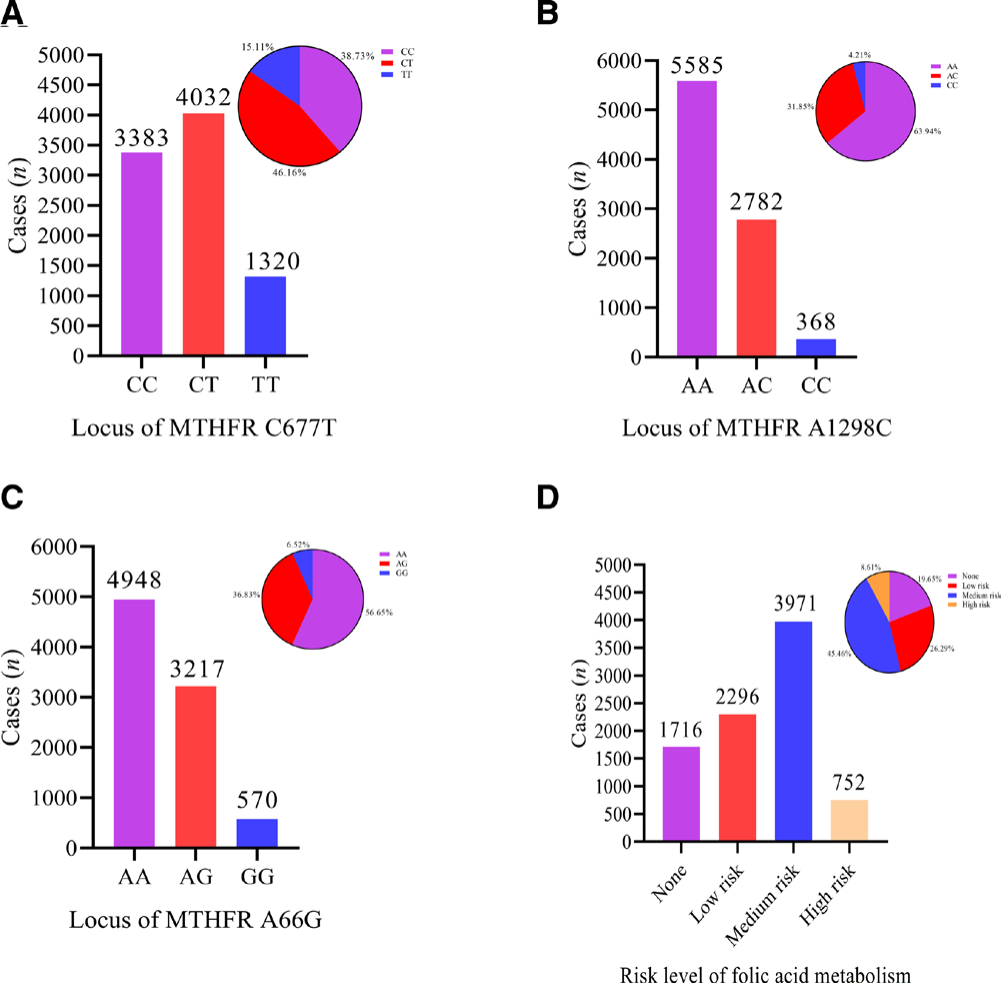
SNPs and risk levels of folate metabolism. (A) Genotype of MTHFR C677T locus in included cases; (B) Genotype of MTHFR A1298C locus in included cases; (C) Genotype of MTRR A66G locus in included cases; (D) Distribution of risk levels for folate metabolism in included case. MTHFR = methylenetetrahydrofolate reductase, MTRR = methionine synthase reductase, SNPS = single nucleotide polymorphisms.

Genotype analysis and allele distribution frequency showed that in the MTHFR C677T locus, there were 3383 cases of pregnant women with CC genotype, 4032 cases with CT genotype, and 1320 cases with TT genotype (Fig. 2A). The frequency of allele C was 61.81%, and allele T was 38.19%. In the MTHFR A1298C locus, there were 5585 cases of AA genotype, 2782 cases of AC genotype, and 368 cases of CC genotype (Fig. 2B), the frequency of allele A was 79.86%, C was 20.14%. In the MTRR A66G locus, there were 4948 cases of AA genotype, 3217 cases of AG genotype, and 570 cases of GG genotype (Fig. 2C). The frequency of allele A was 75.06%, and allele G was 24.94%. In the detection of risk level for folate metabolism, a total of 1716 cases of folate metabolism risk were not found, a total of 2296 cases of low risk level of folate metabolism, a total of 3971 cases of medium risk, and a total of 752 cases of high risk (Fig. 2D). Overall, those results indicated that the included 8735 pregnant women had a relative higher proportion of the risk levels for folate metabolism.

### 3.3. Hardy–Weinberg equilibrium (HWE)

The results of Goodness-of-fit test based on the Chi-square showed that the genotype frequency distribution of MTHFR C677T, MTHFR A1298C, and MTRR A66G conformed to HWE (Table 1), and the difference was not statistically significant (*P* > .05), which demonstrated that the study objects were community representative. The predicted frequencies of CC, CT, and TT genotype of MTHFR C677T were 39%, 46%, and 15%, respectively, and the actual frequencies were 38.73%, 46.16%, and 15.11%, respectively. The predicted frequencies of the AA, AC, and CC genotypes of the MTHFR A1298C were 64%, 32%, and 4%, respectively, and the actual frequencies were 63.94%, 31.85%, and 4.21%, respectively. The predicted frequencies of the AA, AG, and GG genotypes of the MTRR A66G locus were 57.2%, 37.2%, and 7%, respectively, and the actual frequencies were 56.65%, 36.83%, and 6.52%, respectively. In the MTHFR C677T locus, the CC genotype frequency was the highest. The AA genotype frequency was the highest in the MTHFR A1298C and MTRR A66G locus. See Table 1, which illustrates the HWE results of MTHFR (C677T, A1298C) and MTRR A66G.

**Table 1:** Hardy–Weinberg equilibrium (HWE) results of MTHFR (C677T, A1298C) and MTRR A66G.

Gene	Genotype	Actual frequencies % (n)	Predicted frequencies (%)	*x* ^2^	*P*
MTHFR C677T	CC	38.73 (3383)	39	0.003	.998
	CT	46.16 (4032)	46		
	TT	15.11 (1320)	15		
MTHFR A1298C	AA	63.94 (5585)	64	0.011	.994
	AC	31.85 (2782)	32		
	CC	4.21 (368)	4		
MTRR A66G	AA	56.65 (4948)	57.2	0.027	.987
	AG	36.83 (3217)	37.2		
	GG	6.52 (570)	7		

MTHFR = methylenetetrahydrofolate reductase, MTRR = methionine synthase reductase.

### 3.4. Associations of SNPs with susceptibility and risk levels of folate metabolism

The results of spearman correlation analysis showed that the SNPs of MTHFR C677T was positively correlated with the susceptibility of folate metabolism risk, and the correlation coefficient was the highest (*R* = 0.572, *P* < .001, Table 2). The SNPs of MTRR A66G was positively correlated with the risk levels of folate metabolism and had the highest correlation coefficient (*R* = 0.600, *P* < .001, Table 2). In contrast, the SNPs of MTHFR A1298C was negatively associated with the susceptibility of folate metabolism (*r* = −0.160, *P* < .001, Table 2). See Table 2, which illustrates the Association of SNPs with susceptibility and risk levels of folate metabolism.

**Table 2:** Association of SNPs with susceptibility and risk levels of folate metabolism.

SNPs	Susceptibility of folate metabolism		Risk levels of folate metabolism	
	*r*	*P*	*r*	*P*
MTHFR C677T	0.572	<.001	0.226	<.001
MTHFR A1298C	−0.160	<.001	−0.013	.141
MTRR A66G	0.424	<.001	0.600	<.001

MTHFR = methylenetetrahydrofolate reductase, MTRR = methionine synthase reductase, SNPs = single nucleotide polymorphisms.

### 3.5. Correlation of risk factors with susceptibility and risk levels of folate metabolism

The ages, nationality, gestation, number of pregnancy and comorbidity were selected as the risk factors for the susceptibility and risk levels of folate metabolism based on previous reports and clinical reality. As presented in Table 3, the results showed that all risk factors neither affected the susceptibility nor the risk levels of folate metabolism, and there was no significant correlation (*R* < 0.1 or −0.1, *P* > .05, Table 3). See Table 3, which illustrates the Correlation of risk factors with susceptibility and risk levels of folate metabolism.

**Table 3:** Correlation of risk factors with susceptibility and risk levels of folate metabolism.

Risk factors	Susceptibility of folic acid metabolism		Risk levels of folic acid metabolism	
	*r*	*P*	*r*	*P*
Ages	−0.015	.150	−0.014	.185
Nationality	0.018	.095	<0.001	.982
Gestation	0.009	.387	−0.007	.526
Number of pregnancy	−0.008	.434	−0.003	.810
Comorbidity	−0.005	.641	−0.002	.878

### 3.6. Correlation between risk factors and SNPs

The results of Spearman correlation analysis showed that gestation had a potential positive correlation with the SNPs of MTHFR A1298C locus (*R* = 0.024, *P* = .024, Table 4). Further analysis of the allele frequency distribution of the MTHFR A1298C locus in different gestation showed that the distribution frequency of allele A (wild type) in 2753 women of reproductive age in early pregnancy was 1811 cases, C (mutant type) was 942 cases, and 2917 cases were in the second trimester. The distribution frequency of allele A (wild type) in women of childbearing age was 1844 cases, C (mutant type) was 1073 cases, and the distribution frequency of allele A (wild type) in 3065 cases of late pregnancy was 1930 cases, C (mutant type) was 1135 cases example. It is indicated that with the prolongation of pregnancy time, the distribution frequency of allele C (mutant type) of the MTHFR A1298C locus gradually increases, and the function decreases accordingly, which may lead to the susceptibility of folic acid metabolism. See Table 4, which illustrates the Correlation between risk factors and SNPs.

**Table 4:** Correlation between risk factors and single nucleotide polymorphisms (SNPs).

Risk factors	MTHFR C677T		MTHFR A1298C		MTRR A66G	
	*r*	*P*	*r*	*P*	*r*	*P*
Ages	−0.002	.821	0.004	.689	−0.009	.400
Nationality	0.002	.863	0.020	.059	−0.005	.619
Gestation	−0.004	.699	0.024	.024	−0.019	.072
Number of pregnancy	−0.004	.721	−0.009	.396	0.007	.495
Comorbidity	−0.004	.689	−0.002	.880	−0.001	.918

MTHFR = methylenetetrahydrofolate reductase, MTRR = methionine synthase reductase.

### 3.7. Frequency distribution of SNPs in different ethnic groups

The frequency distribution of SNPs in different ethnic groups was analyzed followed by the SNPs test results in 8735 pregnant women. As shown in Table 5, in Han ethnic group, the frequency of CT genotype was the highest (1594/3509), and the frequency of allele C/T was 4336/2682 among the MTHFR C677T locus. The MTHFR A1298C locus had the highest AA genotype frequency in Han ethnic group (2272/3509), and the A/C allele frequency was 5645/1373. Meanwhile, the frequency of AA genotype at the MTRR A66G locus was the highest frequency in Han ethnic group (1982/3509), and the frequency of A/G allele frequency was 5270/1748. The frequency of CT genotype among the MTHFR C677T locus was the highest in Buyi (809/1770) and Miao (446/1024) and Dong (303/608) ethnic group. Furthermore, the frequency of AA genotype among MTHFR A1298C and MTRR A66G locus also was the highest in Buyi, Miao, and Dong ethnic group (Table 5). See Table 5, which illustrates the Frequency distribution of SNPs in different ethnic groups.

**Table 5:** Frequency distribution of single nucleotide polymorphisms (SNPs) in different ethnic groups.

Nationality	MTHFR C677T (n)			MTHFR A1298C (n)			MTRR A66G (n)		
	CC	CT	TT	AA	AC	CC	AA	AG	GG
Han	1371	1594	544	2272	1101	136	1982	1306	221
Buyi	688	809	273	1141	550	79	994	658	118
Miao	417	446	161	651	330	43	563	382	79
Dong	220	303	85	394	183	31	366	194	48
Tujia	211	280	81	351	190	31	336	198	38
Others	475	599	176	775	427	48	705	479	66

Other ethnic groups were the Yi, Bai, Zhuang, and Chuanqing.

MTHFR = methylenetetrahydrofolate reductase, MTRR = methionine synthase reductase.

### 3.8. Associations of different ethnic groups and risk levels of folate metabolism

**Figure 3.**
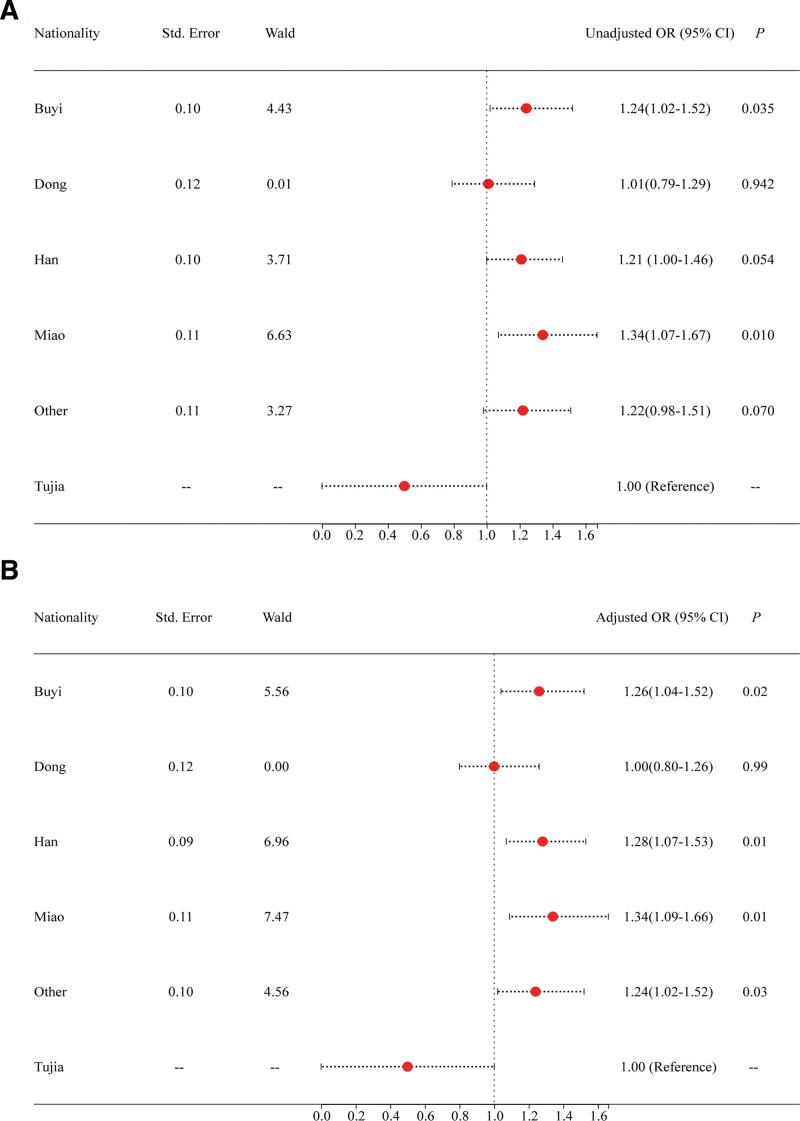
Associations of different ethnic groups and risk levels of folate metabolism. Adjusted for age, nationality, gestation, number of pregnancy and comorbidity. Other ethnic groups were the Yi, Bai, Zhuang, and Chuanqing.

The associations between different ethnic groups and the risk levels of folate metabolism was analyzed by ordinal logistic regression followed by the test of parallel lines (*P* > .05). The results showed that the Buyi (unadjusted OR = 1.24, 95% CI: 1.02–1.52, *P* = .035, Fig. 3A, Table 6) and Miao ethnic group (unadjusted OR = 1.34, 95% CI: 1.07–1.67, *P* = .010, Fig. 3A, Table 6) in pregnant women were significantly related to the risk levels of folic acid metabolism. After adjustment of risk factors (age, nationality, gestation, number of pregnancy and comorbidity), the Buyi (adjusted OR = 1.26, 95% CI: 1.04–1.52, *P* = .02), Han (adjusted OR = 1.28, 95% CI: 1.07–1.53, *P* = .01), Miao (adjusted OR = 1.34, 95% CI: 1.09–1.66, *P* = .01) and other ethnic groups (adjusted OR = 1.24, 95% CI: 1.02–1.52, *P* = .03) were significantly related to the risk levels of folate metabolism (Fig. 3B, Table 6). See Table 6, which illustrates the Associations of SNPs and risk levels of folate metabolism.

**Table 6:** Associations of different ethnic groups and risk levels of folate metabolism.

Nationality	Unadjusted OR (95% CI)	*P*	Adjusted OR* (95% CI)	*P*
Buyi	1.24 (1.02–1.52)	.035	1.26 (1.04–1.52)	.02
Dong	1.01 (0.79–1.29)	.942	1.00 (0.80–1.26)	.99
Han	1.21 (1.00–1.46)	.054	1.28 (1.07–1.53)	.01
Miao	1.34 (1.07–1.67)	.010	1.34 (1.09–1.66)	.01
Others	1.22 (0.98–1.51)	.070	1.24 (1.02–1.52)	.03
Tujia	1.00 (Reference)	--	--	--

OR = odds ratio.

* Adjusted for age, gestation, number of pregnancy, and comorbidity.

### 3.9. Associations of SNPs and risk levels of folate metabolism

Logistic regression analysis showed that the CC genotype of MTHFR C677T locus was significantly related to the risk levels of folate metabolism (unadjusted OR = 5.21, 95% CI: 4.27–6.37, *P* < .001, Table 7). After adjustment of risk factors (age, nationality, gestation, number of pregnancy and comorbidity), the CC genotype of MTHFR C677T locus (adjusted OR = 2.46, 95% CI: 2.14–2.84, *P* < .001) and the AG genotype of MTRR A66G locus (adjusted OR = 1.89, 95% CI: 1.61–2.22, *P* < .001) were significantly related to the risk levels of folate metabolism (Table 7), which was the independent risk factor for the risk levels of folate metabolism. However, the AA, AC, and CC genotype of MTHFR A1298C locus were not significantly correlated with the risk levels of folate metabolism (unadjusted/adjusted OR < 1), which was consistent with the previous results in Section 3.4, suggesting that MTHFR A1298C may be a protective factor. See Table 7, which illustrates the Associations of SNPs and risk levels of folate metabolism.

**Table 7:** Associations of single nucleotide polymorphisms (SNPs) and risk levels of folate metabolism.

Genes	Genotypes	Unadjusted OR (95% CI)	*P*	Adjusted OR* (95% CI)	*P*
MTHFR C677T	CC	5.21 (4.27–6.37)	<.001	2.46 (2.14–2.84)	<.001
	CT	0.29 (0.25–0.32)	<.001	0.39 (0.35–0.43)	<.001
	TT	1.00 (Reference)	--	--	--
MTHFR A1298C	AA	0.62 (0.50–0.77)	<.001	0.97 (0.82–1.16)	.754
	AC	0.68 (0.54–0.85)	.001	0.96 (0.80–1.15)	.647
	CC	1.00 (Reference)	--	--	--
MTRR A66G	AA	0.06 (0.05–0.07)	<.001	0.16 (0.14–0.19)	<.001
	AG	1.18 (0.94–1.47)	.148	1.89 (1.61–2.22)	<.001
	GG	1.00 (Reference)	--	--	--

MTHFR = methylenetetrahydrofolate reductase, MTRR = methionine synthase reductase, OR = odds ratio.

* Adjusted for age, gestation, number of pregnancy, and comorbidity.

## 4. Discussion

Folate metabolism and folic acid supplementation in pregnancy are the crucial link of prenatal and postnatal care. Results from this study indicated that the included 8735 pregnant women had a relative higher proportion of the risk levels for folate metabolism. The frequency distribution of SNPs in different ethnic groups revealed that the CT genotype among the MTHFR C677T locus, AA genotype among MTHFR A1298C and MTRR A66G locus were the highest frequency in Han, Buyi, Miao and Dong ethnic groups. Meanwhile, we found the Buyi, Han, Miao ethnic groups were significantly related to the risk levels of folate metabolism. In addition, we revealed that the CC genotype of MTHFR C677T locus and the AG genotype of MTRR A66G locus were significantly related to the risk levels of folate metabolism, which was the independent risk factor for the risk levels of folate metabolism. However, the genotypes of MTHFR A1298C locus were not significantly correlated with the risk levels of folate metabolism, suggesting that MTHFR A1298C may be a protective factor for folate metabolism.

Nowadays, folic acid supplementation is widely recommended in the group of pregnant women with a recommended daily intake of 400 mg during early pregnancy.^[12]^ In addition to folic acid intake, the folate metabolism genes also plays a crucial role in the folate metabolic pathway. The MTHFR gene is located in the autosomal 1p36.3 region and encodes 656 amino acids. Totally, 15 allelic mutation sites have been found in MTHFR, of which 677C/T and 1298A/C are the 2 polymorphisms of the MTHFR gene.^[13,14]^ The C677T locus of the MTHFR gene includes 3 genetypes: CC, CT, and TT. Mutation of C677T could lead to reduced activity of folate metabolism enzyme. The mutation from CC to TT genetype, the enzyme activity will decrease by 70%, and the mutation to CT genetype, it will decrease by 35%.^[15]^ In European populations, women who carry the 677TT/CT MTHFR genotypes are more likely to be exposed to disturbances in folate metabolism and the consequences of incorrect folate processing during pregnancy.^[16]^ MTHFR 1298A→C mutation could cause glutamic acid to be replaced by alanine in the amino acid sequence.^[17,18]^ The MTRR gene is located in the autosomal 5p15.2 to 15.3 region, consisting of 14 introns and 15 exons. MTRR keeps MTR (an enzyme in the synthesis of methionine from homocysteine) active in the catalytic cycle with the assistance of vitamin B12. The 66A→G mutation on the exon causes the corresponding isoleucine to be converted into methionine, which reduces the activity of MTR, plasma folate levels and the remethylation metabolism of homocysteine.^[19,20]^ Several studies have previously investigated the associations between the folic acid metabolic enzymes-related SNPs and pregnancy outcomes. A case-control study has showed that the parental MTHFR 677C > T and 1298A > C was significantly higher in the pregnant woman with miscarriages compared with controls and significantly associated with miscarriages.^[21]^ Another study has indicated the MTHFR(677)C > T genetic polymorphisms (CT or CT/TT genotypes) significantly associated with infant anthropometry at birth and with a higher risk of small-for-gestational-age in female premature infants, even with adequate folate nutritional status.^[22]^ Furthermore, an increased risk of NTDs was verified to be significantly associated with GG genotype and allele G of MTRR A66G locus in a Chinese population.^[23]^ However, the associations between the polymorphisms of MTHFR A1298C and the lower level of folate were inconsistent. Previous study has verified the CC mutant genotype of MTHFR A1298C was higher among preeclampsia women and related to increased neck circumference and hyperhomocysteinemia.^[24]^ Nevertheless, a case–control study from South Korea indicated MTHFR A1298C polymorphisms are not associated with idiopathic recurrent pregnancy loss in Korean women, indicating that the mutations of MTHFR A1298C may not be susceptible allelic variants or be deficient to cause unfavorable pregnancy outcomes.^[25]^ In our study, we found the SNPs of MTHFR C677T was positively correlated with the susceptibility of folate metabolism risk, and the MTRR A66G was positively correlated with the risk levels of folate metabolism. Furthermore, the CC genotype of MTHFR C677T locus and the AG genotype of MTRR A66G locus were significantly related to the risk levels of folate metabolism, which was the independent risk factor for the risk levels of folate metabolism. In contrast, the SNPs of MTHFR A1298C was negatively associated with the susceptibility of folate metabolism and was not significantly correlated with the risk levels of folate metabolism. Accordingly, in the pregnant woman who carries the mutant CC genotype of MTHFR C677T and the AG genotype of MTRR A66G, the regular monitoring of plasma folate and folate metabolic levels is necessary.

Although the SNPs of folate metabolic genes was one of the important factors in folate utilization, other potential factors also could lead to folic acid metabolism disorder. Age, gestation, number of pregnancy and complications have been reported as the influencing factors of folate metabolism in women of childbearing age.^[26]^ A study conducted by NILSEN et al showed that with the increase in the number of pregnancy, the capacity of folate metabolism decreased.^[27]^ Similar results were obtained in a study conducted by MOSER et al.^[28]^ Conversely, in our study, the risk factors, including age, nationality, gestation, number of pregnancy and comorbidity, were no significant correlation with the susceptibility and the risk levels of folate metabolism. We speculate that the possible reasons could be attributed to the age distribution is mainly concentrated in 26 to 35 years old (average age: 28.87 ± 4.20 years old), and the number of pregnancy is mainly distributed in 1 to 2 times. There is no significant correlation with the susceptibility of folate metabolism may due to the pregnant woman with relative stronger ability for folic acid metabolism.

In conclusion, this study indicates that SNPs of folate metabolism genes, including MTHFR C677T and MTRR A66G, have a significant association with the risk levels of folate metabolism. In addition, we identifies that the Buyi, Han, Miao ethnic groups in our region were significantly related to the risk levels of folate metabolism. Therefore, the detection SNPs of folate metabolism genes and routine assessment of folate metabolism ability for specific populations are particularly necessary. However, our study only included pregnant women in Guiyang and its surrounding areas, which may increase the selection bias of the sample. In addition, there are many ethnic minorities in China, and only a few nationality (Han, Buyi, Miao, Dong, Tujia) are included in this study, which may reduce the representativeness of the sample. Therefore, based on the results of our study, it is necessary to carry out cross-sectional study with multi-regions, multi-centers and large samples in the future.

## Acknowledgments

Our greatest Acknowledgments is to all our colleagues in this study for their hard work. All authors would like to thank all the researchers of the studies in references.

## Author contributions

**Data curation:** Dongyang Deng.

**Writing – original draft:** Hua Huang, Jiangyan He.

**Writing – review & editing:** Rong Chen, Yiyuan Zhou.

## Supplementary Material


