# Supplementary material for: Correlation between single nucleotide polymorphisms of folate metabolism genes and ethnic distribution in pregnant women
Source: Medicine (Baltimore). 2023 Jul 28;102(30):e34472. doi: 10.1097/MD.0000000000034472 (PMC10378908; doi:10.1097/MD.0000000000034472)
Supplement: Supplementary file 1 [file medi-102-e34472-s001.pdf]

贵州中医药大学第一附属医院伦理委员会

伦理审查批件（科研）

<table>
<tr><td>批件号</td><td colspan="3">K2022-011</td></tr>
<tr><td>项目名称</td><td colspan="3">贵阳地区育龄妇女叶酸代谢关键基因位点多态性特征研究及民族分布特征分析</td></tr>
<tr><td>项目来源</td><td colspan="3">论文设计相关研究申请</td></tr>
<tr><td>申请人</td><td colspan="3">周意园、黄华</td></tr>
<tr><td>审查日期</td><td>2022.05.20</td><td>审查地点</td><td>二院区 6 号楼 429 会议室</td></tr>
<tr><td>审查文件</td><td colspan="3">贵阳地区育龄妇女叶酸代谢关键基因位点多态性特征研究及民族分布特征分析</td></tr>
<tr><td colspan="4">审查意见：
根据卫计委《涉及人的生物医学研究伦理审查办法（2016）》、WMA《赫尔辛基宣言》和 CIOMS《人体生物医学研究国际道德指南》的伦理原则，经本伦理委员会审查，同意开展本项科研研究。
请遵循伦理委员会批准的方案开展研究，保护受试者的健康与权力。</td></tr>
<tr><td colspan="2">伦理委员会（盖章）</td><td colspan="2">贵州中医药大学第一附属医院 伦理委员会</td></tr>
<tr><td colspan="2">日期</td><td colspan="2">2022.05.20</td></tr>
</table>
